# The Role of SLC7A11 in Cancer: Friend or Foe?

**DOI:** 10.3390/cancers14133059

**Published:** 2022-06-22

**Authors:** Sijia Li, Zhenyao Lu, Runbin Sun, Suhan Guo, Fangfang Gao, Bei Cao, Jiye Aa

**Affiliations:** 1 China Pharmaceutical University Nanjing Drum Tower Hospital, Nanjing 210000, China; lisijia@suda.edu.cn; 2 Department of Pharmacy, The First Affiliated Hospital of Soochow University, Suzhou 215006, China; 3 Key Laboratory of Drug Metabolism and Pharmacokinetics, China Pharmaceutical University, Nanjing 210009, China; 3119070213@stu.cpu.edu.cn (Z.L.); 3121070239@stu.cpu.edu.cn (S.G.); 3220070806@stu.cpu.edu.cn (F.G.); 4 Phase I Clinical Trials Unit, Nanjing Drum Tower Hospital, The Affiliated Hospital of Nanjing University Medical School, Nanjing 210008, China; runbinsun@cpu.edu.cn

**Keywords:** SLC7A11, tumour, tumourigenesis, survival and proliferation, metastasis, therapeutic resistance

## Abstract

**Simple Summary:**

Strikingly, there are many literature reports confirming that the SLC7A11 gene is closely related to the tumourigenesis, survival, proliferation, metastasis, therapeutic resistance, and other aspects of tumour cells. Meanwhile, the role of SLC7A11 in tumours is highly complex, and its pro-tumour and antitumour activities are obviously different. We summarised the biological characteristics of SLC7A11, including its structure, expression, function, regulation, and therapeutic approaches, and focused on the discussion and analysis of the possibility of SLC7A11 as a potential antitumour target, providing a theoretical basis for drug research and clinical tumour treatment.

**Abstract:**

SLC7A11 controls the uptake of extracellular cystine in exchange for glutamate at a ratio of 1:1, and it is overexpressed in a variety of tumours. Accumulating evidence has shown that the expression of SLC7A11 is fine-tuned at multiple levels, and plays diverse functional and pharmacological roles in tumours, such as cellular redox homeostasis, cell growth and death, and cell metabolism. Many reports have suggested that the inhibition of SLC7A11 expression and activity is favourable for tumour therapy; thus, SLC7A11 is regarded as a potential therapeutic target. However, emerging evidence also suggests that on some occasions, the inhibition of SLC7A11 is beneficial to the survival of cancer cells, and confers the development of drug resistance. In this review, we first briefly introduce the biological properties of SLC7A11, including its structure and physiological functions, and further summarise its regulatory network and potential regulators. Then, focusing on its role in cancer, we describe the relationships of SLC7A11 with tumourigenesis, survival, proliferation, metastasis, and therapeutic resistance in more detail. Finally, since SLC7A11 has been linked to cancer through multiple approaches, we propose that its contribution and regulatory mechanism require further elucidation. Thus, more personalised therapeutic strategies should be adapted when targeting SLC7A11.

## 1. Introduction

SLC7A11 (solute carrier family 7 member 11), which is also known as xCT or CCBR1, encodes a member of a heteromeric, sodium-independent, anionic amino acid transport system that is highly specific to cystine and glutamate [1]. xCT is a light chain that is linked by a disulphide bond (-S-S-) to the 4F2 heavy chain (4F2hc, also called CD98 or SLC3A2) to constitute the x_c_− cystine/glutamate antiporter (system x_c_−), which belongs to the family of heterodimeric amino acid transporters (HATs) [2]. Together, the two subunits are in charge of amino acid transportation. SLC7A11 is responsible for the transport activity. 4F2hc is a regulatory subunit responsible for trafficking of the light chain, and is required for cell surface expression [3]. Since various studies indicate that 4F2hc does not seem to be prominently involved in the regulation of system x_c_− activity, and quite a few members in the HAT family also contain 4F2hc, the component SLC7A11 is considered to be the functional unit, and is credited with the specificity of system x_c_− [4].

SLC7A11 imports the amino acid cystine, and cystine and cysteine can interconvert. Cysteine serves as a crucial and even rate-limiting substrate for glutathione (GSH) synthesis and, thus, plays an important role in oxidative protection [5]. Emerging evidence also shows that SLC7A11 can act on its own as a GSH-independent redox system by sustaining a redox cycle over the plasma membrane that is required for proper cell signalling and communication [6]. This cycle is marked by cystine uptake, intracellular reduction to cysteine, and secretion of cysteine to provide a reducing microenvironment for appropriate cellular signalling, e.g., T-cell activation [7]. Furthermore, it also mediates the export of glutamate, which is involved in many physiological and biochemical processes. For example, glutamate is a major source of nitrogen and energy for dividing cells, including cancer cells, and it is an abundant amino acid in most mammalian tissues [8,9,10]. Glutamate could also act as an excitatory neurotransmitter and participate in either neuronal signalling or excitotoxic pathology [4,11,12]. Considering that oxidative stress and the disturbance of glutamate have been linked to a wide range of diseases and treatments in multiple ways, it can be readily inferred that SLC7A11 can participate in various diseases.

To date, continuing evidence has proven that SLC7A11 is closely related to various aspects of cancer, including tumourigenesis, proliferation, metastasis, prognosis, and chemoresistance. Nevertheless, a high degree of complexity has emerged regarding the role of SLC7A11 in cancer, with clear discrepancies between pro- and antitumourigenic activities evident when utilising cell culture versus in vivo models of malignancy. Furthermore, SLC7A11 is a potentially important drug target that can be effectively modulated with different classes of compounds. In the present review, we briefly introduce the biological properties of SLC7A11. We primarily focus on what kind of relationship exists between SLC7A11 and cancer, and how they are connected. The potential for the therapeutic modulation of SLC7A11 activity in cancer is also discussed.

## 2. Biological Properties

System x_c_− was first characterised in human foetal lung fibroblasts in culture by Bannai and Kitamura in 1980 [13]. cDNA encoding the transporter for system x_c_− was first isolated by Sato et al. from murine activated macrophages via expression in *Xenopus* oocytes in 1999 [1]. They identified the promiscuous 4F2hc as one subunit, and a new 502 amino acid protein named SLC7A11 as the specific light chain subunit of system x_c_−. Subsequently, they isolated two cDNAs encoding SLC7A11 from the human cDNA library, with one clone encoding a protein of 501 amino acids with 12 putative transmembrane domains [14]. Continual research on this topic has been carried out in subsequent years, and achievements have been made. Its biological properties—including structure, expression, functional and pharmacological roles, regulation, and therapeutic approaches—are briefly described in this section.

### 2.1. Structure

To date, 506 SLC7A11 genes have been identified in the PubMed gene database. The SLC7A11 gene is conserved in chimpanzees, rhesus monkeys, dogs, cows, mice, rats, chickens, zebrafish, and frogs, and 209 organisms have orthologues with the human gene SLC7A11. The human SLC7A11 gene (Gene ID: 23657) is localised at chromosome 4q28.3, and the reference cDNA (NM_014331.3) published online is 9648 bp long, with a 280 bp 5′UTR and a 7862 bp 3′UTR. Cloning of human *SLC7A11* from cDNA libraries of different cells—including W126Va4 cells, the human retinal pigment epithelial cell line ARPE-19, the human teratoma cell line NT2, and human glioma U87 cells—has yielded putative transcripts of different lengths [14,15,16,17]. All of these cDNA sequences share a 231 bp 5′UTR and a 1506 bp open reading frame (ORF), including the stop codon. However, they have divergent 3′UTRs. Structural studies, including those based on cysteine scanning and thiol-modifying reagent accessibility, indicate that the SLC7A11 protein has 12 transmembrane domains (TMDs), intracellular N and C termini, and a re-entrant loop between TMDs 2 and 3 that appears to participate in substrate binding and/or permeation [18,19].

### 2.2. Expression

RNA-Seq data from 95 human individuals demonstrated that SLC7A11 shows a tissue-specific distribution, with the normalised mRNA abundance varying greatly among 27 different tissues [20]. The quantitative FPKM values (the transcriptome of each sample was quantified using RNA-Seq to determine the normalised mRNA abundance, calculated as FPKM values) indicated that the mRNA expression of SLC7A11 was highest in the brain, followed by the thyroid > stomach > appendix > urinary bladder > gall bladder, and low in the kidneys, heart and liver. These findings are largely consistent with the RNA sequencing results of total RNA from 20 human tissues [20]. In vivo, SLC7A11 has a rather restricted expression pattern, with the highest levels in the central nervous system (CNS) [1,21] and parts of the immune system, such as antigen-presenting cells (APCs) and myeloid-derived suppressor cells (MDSCs) [22,23]. The expression levels of the SLC7A11 gene and protein were increased in M2 macrophages that were induced by IL-4 in a murine model [24]. Interestingly, the relative expression of the different *SLC7A11* mRNA transcripts also shows tissue specificity, with the 12 kb form being much more abundant in the brain and meninges than in macrophages, where the 2.5 and 3.5 kb forms are more prominently expressed [25].

Considering the localisation and functional importance of SLC7A11, it is not surprising that it could be involved in diseases such as psychiatric and neurodegenerative diseases, eye diseases, and immune response, as has been indicated by continuously emerging reports. For example, the expression of SLC7A11 in peripheral blood and postmortem optic nerve samples from multiple sclerosis (MS) patients was assessed by quantitative PCR. The results showed that there was a significant increase in SLC7A11 mRNA expression in leukocytes in relapsing MS—which is more prominent during relapses—and a higher expression of SLC7A11 in the CNS of MS patients than in controls [26]. Notably, SLC7A11 has been frequently observed in various malignant tumours, including lymphomas, leukaemias, Kaposi’s sarcoma, squamous-cell carcinomas/epithelial carcinomas, breast cancer, glioblastoma (GBM), and pancreatic cancers (PDAC) [4,27,28]. Moreover, it has also been shown to be expressed in various lung [29], prostate [30], ovarian [31], bladder [32], gastric [33], and breast cancer cell lines [34]. We further expand on the role of SLC7A11 in cancer and its potential to serve as a druggable target for anticancer therapy in the following sections.

### 2.3. Functional and Pharmacological Roles

SLC7A11 transports cystine into cells in exchange for glutamate at a ratio of 1:1. In general, the functions of SLC7A11 can be classified as direct and indirect effects. Both cystine and glutamate are important endogenous substances that can produce pharmacological effects directly. An indirect function comes from biotransformation products using glutamate or cystine as substrates, since the two substrates can participate in other metabolic pathways to generate bioactive metabolites with multiple biological and pharmacological roles.

First, extracellular glutamate can be transported into cells by the glutamate transporter family [35]. In particular, Na^+^-dependent excitatory amino acid transporters are responsible for the majority of glutamate reuptake within the CNS [36,37], while in glial cells, the glutamate–glutamine cycle exists. In this cycle, glutamate is converted to glutamine and subsequently transported back into presynaptic neurons. It is then converted back into glutamate and taken up into synaptic vesicles [38,39]. Based on the literature, an imbalance in glutamate homeostasis plays a critical role in GBM [40], addiction [41], glioma-related seizures [42], coronary heart disease [43], multiple sclerosis [44,45], and depression [46]. For example, SLC7A11 plays a crucial pharmacological role in tumours—especially in CNS tumours. Upregulation of SLC7A11 increases glutamate secretion in patients with GBM. This results in the disturbance of glutamate homeostasis, and thereby affects the tumour microenvironment [47]. Such a disturbance can lead to nerve damage and brain swelling, which may be a direct consequence of the tumour microenvironment [48]. Several excellent reviews describing CNS damage by glutamate are available, and we do not discuss these topics in detail [4,49]. Moreover, glutamine plays an essential role in glutamate-related metabolism [50]. Extracellular glutamine is imported into cells by alanine–serine–cysteine transporter 2 (ASCT2), and then converted to glutamate by glutaminase (GLS). The resulting glutamate is converted by glutamate dehydrogenase (GDH) to α-ketoglutarate (α-KG) in the mitochondria to affect the tricarboxylic acid cycle (TCA cycle) and oxidative phosphorylation (OXPHOS) [51,52].

**Figure 1:**
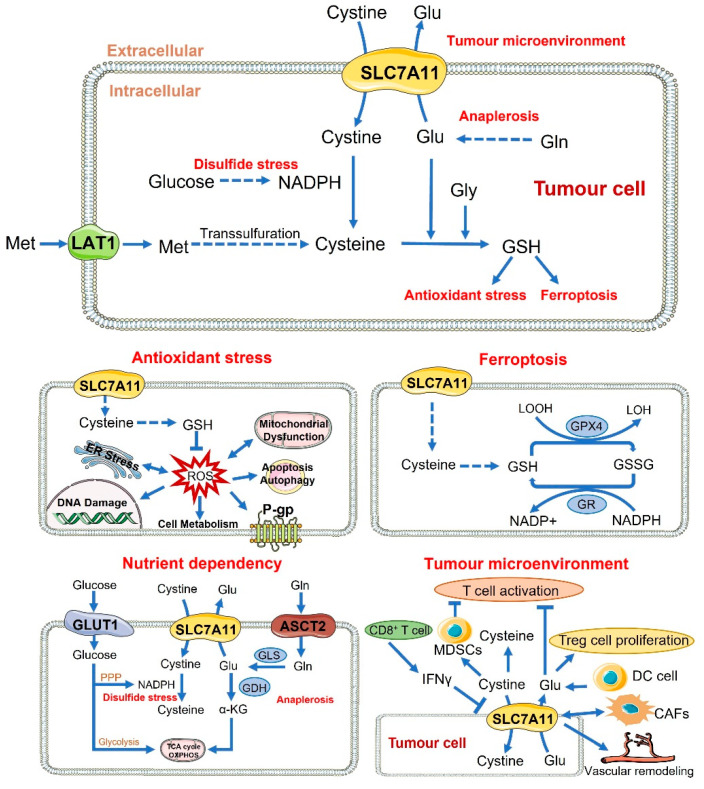
Functional and pharmacological roles of SLC7A11 in cancer: The function of SLC7A11 in cancer can be divided into four aspects: antioxidant function, ferroptosis, nutrient dependency, and tumour microenvironment. SLC7A11, as a cystine/glutamate antiporter, imports cystine and mediates the export of glutamate at a ratio of 1:1. Cysteine is also formed by methionine through trans-sulphuration. Cysteine synthesises glutathione with glutamate and glycine, and participates in oxidative-stress-related processes, such as ferroptosis, apoptosis, autophagy, mitochondrial dysfunction, ER stress, DNA damage, and drug resistance. SLC7A11 is involved in disulphide stress and anaplerosis to make tumour cells become nutrient dependent. Disulphide stress: the cystine transported by SLC7A11 is reduced to cysteine under the action of NADPH, most of which is produced by the pentose phosphate pathway. Anaplerosis: Extracellular glutamine is taken up into the cells and converted to glutamate by GLS. Glutamate is converted into α-KG under GDH, and participates in the TCA cycle and oxidative phosphorylation. The amount of cysteine and glutamate in the tumour microenvironment could affect the survival, proliferation, activation, and function of various immune cells, such as T cells, Treg cells, MDSCs, and DC cells. Moreover, cytokines released by immune cells could affect the expression and function of SLC7A11 in tumour cells. SLC7A11 might be related ro the CAFs or vascular remodelling. ASCT2: alanine–serine–cysteine transporter 2; CAFs: cancer-associated fibroblasts; DC cell: dendritic cell; ER stress: endoplasmic reticulum stress; GDH: glutamate dehydrogenase; Gln: glutamine; GLS: glutaminase; Glu: glutamate; GLUT1: glucose transporter type 1; Gly: glycine; GPX4: glutathione peroxidase 4; GR: glutathione reductase; GSH: reduced glutathione; GSSG: oxidised glutathione disulphide; IFN-γ: interferon γ; LAT1: L-type amino acid transporter 1; LOH: lipid alcohol; LOOH: lipid hydroperoxide; MDSCs: myeloid-derived suppressor cells; Met: methionine; NADP+: oxidised nicotinamide adenine dinucleotide phosphate; NADPH: reduced nicotinamide adenine dinucleotide phosphate; OXPHOS: oxidative phosphorylation; P-gp: P-glycoprotein; PPP: pentose phosphate pathway; ROS: reactive oxygen species; SLC7A11: solute carrier family 7 member 11; TCA cycle: tricarboxylic acid cycle; Treg cell: regulatory T cell; α-KG: α-ketoglutarate.

Second, the growth and progression of cancers have a critical growth requirement for extracellular cystine/cysteine. Cystine is one of the few amino acids that contain sulphur. Imported cystine can be used to produce another amino acid—taurine—and can also be converted into glucose as a source of energy. The reduction of one molecule of cystine to two molecules of cysteine requires the involvement of nicotinamide adenine dinucleotide phosphate (NADPH), produced by the pentose phosphate pathway. In addition, cystine may also play an important role in the communication between immune system cells [53,54,55], aid in the supply of insulin to the pancreas [56], and supply enough substrate for pheomelanin synthesis [57]. Importantly, it is necessary to maintain GSH levels by reducing cysteine, which is the rate-limiting substrate for GSH synthesis. GSH is required for cell survival and proliferation, redox cycling, antioxidative defence, detoxification, drug resistance, and the immune response [58,59,60,61]. In other words, cystine functions as an antioxidant, and is an important part of redox systems, playing an essential role in balancing the levels of reactive oxygen species (ROS). ROS and ROS-mediated signalling are involved in several cellular and biochemical processes, including apoptosis, ferroptosis, autophagy, cell proliferation and migration, endoplasmic reticulum stress, mitochondrial dysfunction, DNA damage, cell metabolism, and drug resistance [62,63,64,65,66,67]. All of these processes are deeply related to the occurrence, development, treatment, and prognosis of cancer. In conclusion, there seems to be a broad and complicated link between SLC7A11 and cancer (Figure 1).

### 2.4. Regulation

**Figure 2:**
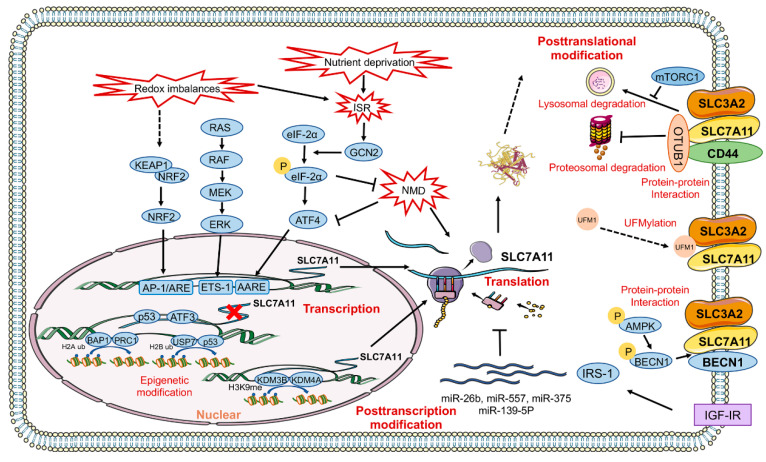
Regulation of SLC7A11 gene expression at multiple levels: Transcription levels: KEAP1-NRF2-AP-1/ARE axis, GCN2-eIF-2α-ATF4 axis, RAS-RAF-MEK-ERK-ETS-1 axis, p53, and ATF3. Epigenetic modification levels: BAP1- and PRC1-associated histone H2A ubiquitination, USP7- and p53-associated histone H2B ubiquitination, and KDM3B- and KDM4A-associated histone methylation. Posttranscriptional levels and translational levels: NMD, a series of microRNAs, and IGF-IR-IRS-1. Posttranslational levels: Protein degradation: the mTORC1-lysosomal degradation pathway and OTUB1-ubiquitin-proteasomal degradation pathway. Protein modification: UFM1-UFMylation. Protein–protein interactions: SLC7A11 and CD44, SLC7A11 and BECN1. AARE: amino acid response element; AMPK: adenosine 5‘-monophosphate-activated protein kinase; AP-1: activator Protein-1; ARE: antioxidant response element; ATF3: activating transcription factor 3;ATF4: activating transcription factor 4; BAP1: BRCA1-associated protein-1; BECN1: beclin 1; CD44: CD44 molecule; eIF-2α: eukaryotic initiation factor 2α; GAS: gamma-activated sequence; GCN2: general control nonderepressible-2; H2A ub: histone H2A ubiquitination; H2B ub: histone H2B ubiquitination; H3K9me: methylation of histone H3 Lys 9; IGF-IR: type I insulin-like growth factors receptor; IRS-1: insulin receptor substrate; KDM3B: histone H3 lysine 9 demethylase 3B; KDM4A: histone H3 lysine 9 demethylase 4A;KEAP1: Kelch-like ECH-associated protein-1; mTORC1: mechanistic target of rapamycin complex 1; NMD: nonsense-mediated mRNA decay; NRF2: nuclear factor erythroid 2-related factor 2; OTUB1: OTU deubiquitinase, ubiquitin aldehyde binding 1; p53: tumour protein p53; PRC1: polycomb repressive complex 1; SLC3A2: solute carrier family 3 member 2; SLC7A11: solute carrier family 7 member 11; STATs: signal transducer and activator of transcription; UFM1: the ubiquitin-fold modifier 1; USP7: ubiquitin-specific processing protease 7.

Determining the function of genes and revealing their regulatory mechanisms can benefit our understanding of the essence of life, and is especially helpful in pathology and drug development. In this section, we summarise the influences of SLC7A11, and focus on expounding its molecular regulatory mechanisms. Here, through the investigation of a flood of literature, the regulatory mechanism of SLC7A11 was mapped out at the DNA level, transcription level (i.e., gene activation and transcription initiation), posttranscriptional level (i.e., processing and transport), translational level, and posttranslational level, with the transcriptional level of regulation being the most important (Figure 2). The Kelch-like ECH-associated protein 1 NFE2-like bZIP transcription factor 2-activator protein-1/antioxidant response element (KEAP1-NRF2-AP-1/ARE) signalling pathway is the most researched and best-characterised mechanism involved in the regulation of SLC7A11 [29,32,47,58,68,69,70]. Redox imbalances stimulate the degradation of the KEAP1–NRF2 complex, and NRF2 is transferred freely to the nucleus. There, it acts on AP-1/ARE, and increases the transcription of genes that resist oxidative stress, including SLC7A11 [5]. Nutritional deficiency or redox imbalances can cause the integrated stress response (ISR) of cells, and stimulates the general control nonderepressible-2-eukaryotic initiation factor 2α—activating the transcription factor 4 (GCN2-eIF-2α-ATF4) pathway, and driving the amino acid response element (AARE) to increase SLC7A11 transcription [71,72,73]. The ETS-1 transcription factor downstream of the RAS-RAF-MEK-ERK signalling cascade directly transactivates the SLC7A11 promoter in synergy with ATF4 [74]. The p53 protein targets the SLC7A11 gene in transcriptional repression by serving as a DNA-binding transcription factor [75], and activating transcription factor 3 (ATF3) represses SLC7A11 expression by binding to the SLC7A11 promoter in a p53-independent manner [76]. Epigenetic changes also regulate the transcription of SLC7A11; such changes include alterations to BRCA1-associated protein-1 (BAP1), polycomb repressive complex 1 (PRC1)-associated histone H2A ubiquitination [77], ubiquitin-specific processing protease 7 (USP7), p53-associated histone H2A ubiquitination [78], KDM3B histone H3 lysine 9 demethylase 3B (KDM3B), and histone H3 lysine 9 demethylase 4A (KDM4A)-associated histone methylation [79,80]. Nonsense-mediated mRNA decay (NMD) [81] and a series of microRNAs [82,83,84,85] affect SLC7A11 protein expression by modulating the posttranscriptional and translational processes. Moreover, type I insulin-like growth factor (IGF-I) could regulate SLC7A11 protein expression in an insulin receptor substrate 1 (IRS-1)-dependent manner [86].

Recently, studies have found that the posttranslational regulation of SLC7A11 modulates downstream biological effects (Figure 2). The SLC7A11 protein could be degraded by autophagy and the ubiquitin–proteasome system. High cell density inactivates mammalian target of rapamycin (mTOR) and promotes the lysosomal degradation of SLC7A11, leading to improved GBM cell viability under glucose-limited conditions [87]. mTOR phosphorylates and sequesters helix–loop–helix transcription factor EB to inhibit lysosomal gene expression [88,89], and controls lysosomal functions through ATP-sensitive Na+ channels [90]. Although there is a close relationship between mTOR activity and lysosomal function, how mTOR is regulated and how SLC7A11 is delivered to lysosomes in response to cell density in GBM cells should be investigated in future studies. The tumour stem cell molecules CD44 [91,92,93] and OTU deubiquitinase ubiquitin aldehyde binding 1 (OTUB1) [94] were beneficial to the stability of SLC7A11 on the cell membrane, and inhibited the proteasomal degradation of SLC7A11. Protein–protein interactions also regulate SLC7A11 function. Beclin 1 (BECN1) inhibits system x_c_− activity through direct binding to SLC7A11, but not through SLC7A11 expression [95]. SLC7A11 is a new UFMylation substrate, and ubiquitin-fold modifier 1 (UFM1) increases its protein stability in a posttranscriptional manner. UFM1/SLC7A11 may be a new potential anticancer target, but how UFM1 acts on SLC7A11 remains an unanswered question [96].

### 2.5. Therapeutic Approaches of SLC7A11

SLC7A11 is known to be strongly induced by various stimuli, such as oxidative stress, amino acid deprivation, and xenobiotic exposure, while quite a few drugs—especially anticancer drugs—have been shown to trigger the downregulation of SLC7A11 [34,97]. Many therapeutic approaches have been demonstrated in preclinical models to modulate the activity or expression of SLC7A11 in cancer. Cystine and glutamate could be both substrates and regulators of SLC7A11. Adequate cystine supplementation in tumour-bearing mice with breast cancer increased the expression of SLC7A11 and promoted the synthesis of GSH [34]. High extracellular glutamate inhibits SLC7A11 and depletes intracellular cystine and cysteine [98]. According to the published literature, some drugs have been recorded to target cystine/glutamate transporters, and their actions are listed in Table 1. Acetylcysteine is a precursor of cysteine and an activator of SLC7A11 [99]. Riluzole, an inhibitor of glutamatergic signalling, inhibits the expression of the cystine–glutamate amino acid antiporter in human melanoma cell lines, and reduces tumour proliferation [100]. Pharmacological agents that inhibit SLC7A11 activity with high potency have long been sought. Sulfasalazine is currently one of the most widely used SLC7A11 inhibitors in the laboratory, and more potent compounds based on its scaffold have been used for development [101,102]. It can significantly inhibit cystine and GSH synthesis, and inhibits tumour growth in both in vitro and in vivo models [103]. Erastin and its analogues trigger ferroptosis, and their inhibitory effect is over 1000 times more potent than SASP [104]. Some approved drugs, such as sorafenib and lanperisone, have also been found to inhibit the activity of SLC7A11, and have certain antitumour effects [104,105]. However, recent studies have shown that sorafenib may not qualify as a bona fide SLC7A11 inhibitor. This inhibitory action can only be achieved in a fraction of tumour cell lines [106], and the effective concentrations for inducing ferroptosis only include a narrow range of concentrations [104]. Although approved drugs such as SASP have been demonstrated to have good pharmacological effects on SLC7A11, there are no clinical studies on their tumour-suppressive effects. In addition to endogenous substances and drugs, there are other ways to target SLC7A11. The ablation of CD44 destroyed the stability of SLC7A11 at the posttranslational level in a transgenic murine model of gastric cancer [91]. Treatment with the human cyst(e)inase enzyme selectively depletes cysteine and cystine in cancer cells, and it inhibits both prostate and breast cancer xenografts’ tumour growth due to depletion of intracellular GSH and the ensuing elevated ROS [107]. SLC7A11 immunotargeted therapies—such as the SLC7A11-targeted DNA vaccination [108], a virus-like particle immunotherapy targeting the SLC7A11 protein [109], and an anti-SLC7A11 viral vaccine based on the bovine herpesvirus 4 vector [110]—also impede the progression of breast cancer by inhibiting SLC7A11.

## 3. The Role of SLC7A11 in Tumours

**Figure 3:**
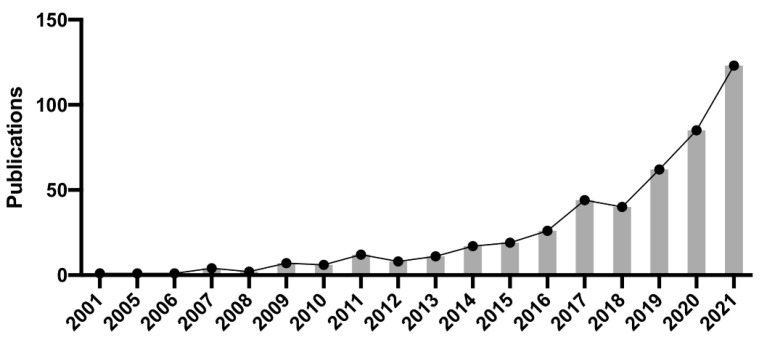
Bibliometric analysis of published literature in the last 20 years, using “SLC7A11” and “Cancer” as search terms.

We searched the Web of Science database to perform bibliometric analysis, using “SLC7A11” and “Cancer” as search terms. The statistical results showed that the number of articles published has increased each year, and the relative research interest has also grown (Figure 3). Highly related concepts involve neoplasms, cell growth and survival, the microenvironment, tumour metastasis, and therapeutic resistance. Thus, these topics are discussed below.

### 3.1. SLC7A11 vs. Tumourigenesis

The SLC7A11 transporter is involved in different mechanisms of multistage reactions during tumourigenesis in different types of cancer cells. Previous studies identified that SLC7A11 is necessary for the initiation and growth of primary pancreatic tumours [116] and melanoma [100]. Similarly, the expression of SLC7A11 was induced by smoking in normal airway epithelial cells, which may be an essential early step in lung tumour initiation [117]. The promotion of SLC7A11-dependent antioxidant function is critical for some tumourigenesis, and blocking this process may benefit future treatments. The combination of the anti-inflammatory drug ibuprofen and the SLC7A11 inhibitor SASP with chemotherapy showed better efficacy, with obvious growth delay of 3-methylcholanthrene (3-MCA) sarcoma, decreased tumour volume, and improved survival rate in mice [103]. In lung adenocarcinoma, YT521-B homology domain containing 2 (YTHDC2), which destabilises SLC7A11 mRNA and blocks the downstream antioxidant program in an m6A (N6-methyladenosine)-dependent manner, frequently suppresses tumourigenesis [118]. RAS upregulates SLC7A11 via RAF-MEK-ERK not only as an adaptive response to oxidative stress, but also as an intrinsic mechanism supporting cellular transformation. Indeed, genetic or pharmacological inhibition of SLC7A11 severely impaired the transformation and tumourigenic potential of oncogenic RAS-expressing cells in vitro and in vivo [74,119].

Conversely, SLC7A11 deficiency sometimes also increases the progression of tumourigenesis. In SLC7A11-deficient mice, overexpression of inflammatory cytokines—such as interleukin-1 (IL-1β) and tumour necrosis factor α (TNF-α)—led to impaired survival of macrophages that were activated in inflammatory sites, sustained inflammation, and an accelerated occurrence of 3-MCA-induced fibrosarcoma [120]. Triple-negative breast cancer (TNBC) cells secrete glutamate, which inhibits SLC7A11 and causes cysteine depletion in cells. EglN1 (the main HIF prolyl-hydroxylase) undergoes oxidative self-inactivation in the absence of cysteine, and directly increases the accumulation of hypoxia-inducible factor (HIF1), while stimulating the orthotopic tumour formation of TNBC [98].

### 3.2. SLC7A11 vs. Survival and Proliferation

#### 3.2.1. Antioxidant Function

**Figure 4:**
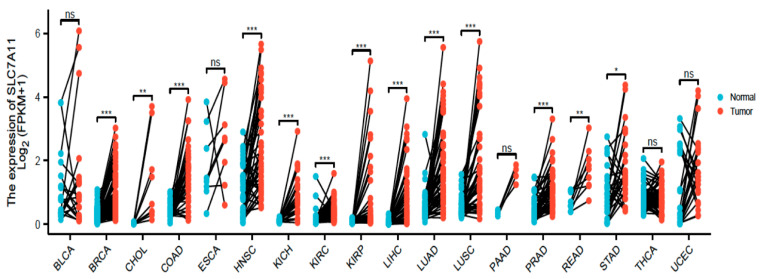
SLC7A11 expression in tumour and normal paired tissues was obtained from pan-cancer data of the Cancer Genome Atlas (TCGA). ns, *p* ≥ 0.05; *, *p* < 0.05; **, *p* < 0.01; ***, *p* < 0.001. BLCA: bladder cancer; BRCA: breast invasive carcinoma; CHOL: cholangiocarcinoma; COAD: colon adenocarcinoma; ESCA: oesophageal carcinoma; HNSC: head and neck squamous-cell carcinoma; KICH: kidney chromophobe; KIRC: kidney renal clear-cell carcinoma; KIRP: kidney renal papillary-cell carcinoma; LIHC: liver hepatocellular carcinoma; LUAD: lung adenocarcinoma; LUSC: lung squamous-cell carcinoma; PAAD: pancreatic adenocarcinoma; PRAD: prostate adenocarcinoma; READ: rectum adenocarcinoma; STAD: stomach adenocarcinoma; THCA: thyroid carcinoma; UCEC: uterine corpus endometrial carcinoma.

One of the most important physiological functions of SLC7A11 is the uptake of cystine, which synthesises GSH and eliminates ROS. Due to genetic changes and the abnormal growth of cancer cells, the oxidative stress caused by cancer cells from ROS is much higher than that of non-malignant cells. Therefore, SLC7A11 is upregulated in many tumour cells compared with paired cells from normal tissue, as evidenced by bioinformatic analysis from The TCGA database (Figure 4). It may be important to develop genetic and pharmacological approaches to inhibit SLC7A11, as summarised in detail in Section 2.5. Such inhibition could cause a reduction in intracellular GSH and a subsequent elevation in ROS. Thus, tumour survival and proliferation would be suppressed by the destruction of the antioxidant function. However, SLC7A11 is not upregulated in all tumour cells, with exceptions including bladder cancer (BLCA), oesophageal carcinoma (ESCA), and uterine corpus endometrial carcinoma (UCEC). Due to the impaired expression of SLC7A11, ARID1A (an SWI/SNF chromatin-remodelling factor)-deficient cancer cells [121] and p53 functional mutant cells [122] have lower basal levels of GSH. Thus, SLC7A11 and oxidative stress may be metabolic vulnerabilities for these cells.

#### 3.2.2. Ferroptosis

Recently, ferroptosis has been suggested to be one of the important mechanisms of tumour inhibition. Ferroptosis is a mechanism of cell death characterised by the accumulation of lipid peroxidation products and ROS from iron metabolism [123]. SLC7A11 is suggested to be involved in ferroptosis. SLC7A11 could synthesise GSH, which is a cofactor to detoxify lipid peroxides, thereby suppressing ferroptosis. SLC7A11-mediated ferroptosis resistance may contribute to proto-oncogene KRAS-driven tumour growth and development [124,125]. Mechanically, KRAS regulates the transcription of SLC7A11 by stimulating the ATF4 endoplasmic-reticulum-stress-associated transcription factor and Nrf2 oxidative-stress-associated transcription factor to preserve intracellular redox balance [74,125]. The deficiency of tumour suppressors, such as p53, BAP1, and ARF, result in SLC7A11 upregulation and ferroptosis resistance [75,77,126]. p53 induces ferroptosis at least in part by inhibiting SLC7A11 expression in NSCLC [75]. BAP1, a nuclear deubiquitase, represses SLC7A11 expression by reducing H2A ubiquitination on the SLC7A11 promoter, thereby regulating ferroptosis [77]. In addition, ARF (an alternative reading frame product of the CDKN2A locus and a tumour suppressor) expression sensitises cells to ferroptosis in a p53-independent manner. ARF inhibits the NRF2 gene to transcriptionally activate its target gene SLC7A11, which could regulate ROS-induced ferroptosis [126]. The N6-methyladenosine (m6A) modification plays a key role in SLC7A11-mediated ferroptosis. The METTL3-mediated m6A modification promotes SLC7A11 mRNA stability and upregulates its expression [127]. NF-κB-activating protein (NKAP)—an RNA-binding protein to m6A—could promote SLC7A11 mRNA splicing and maturation to suppress ferroptosis in GBM [128]. Circular ribonucleic acids (circRNAs) and small-molecule RNA (miRNA) could regulate ferroptosis by affecting posttranscriptional modifications of SLC7A11. The circRNA known as circP4HB acts as a ferroptosis suppressor in LUAD via modulation of the miR-1184/SLC7A11 axis [129]. The miR-34c-3p/SLC7A11 axis is also a potential target to regulate ferroptosis in OSCC [130]. Not only SLC7A11 expression, but also the stabilisation of the SLC7A11 protein could promote tumour growth by inhibiting ferroptosis. CD44 and OTUB1 interact with SLC7A11 to enhance the protein stability and ferroptosis suppression [94]. Therefore, the researchers found that tumour growth could be suppressed by inhibiting SLC7A11 to increase ferroptosis. The pharmacological inhibitors of SLC7A11 and the administration of cyst(e)inase—a drug that depletes cysteine and cystine—are some translatable means to induce ferroptosis [75,104,131].

#### 3.2.3. Nutrient Dependency

Strikingly, SLC7A11, in addition to its well-known antioxidant role, is an important metabolic regulator that affects the nutrient flexibility of cells. Cancer cells with high SLC7A11 expression take up a large amount of cystine into the cell, and the cell rapidly reduces it to cysteine. This reaction requires NADPH, which is mainly provided in the cytoplasm through the glucose–pentose phosphate pathway. Therefore, cells with high SLC7A11 expression show a high dependence on the glucose and pentose phosphate pathways [132], and are more sensitive to glucose-starvation-induced cell death in GBM [133,134]. Co-targeting GLUT1 (glucose transporter type 1) and GSH synthesis may lead to NADPH depletion and the accumulation of ROS. Thus, it could induce synthetic lethal cell death in high-SLC7A11-expressing cell lines susceptible to glucose deprivation [132,135,136].

SLC7A11 also affects the nutritional dependence of tumour cells through glutamine anaplerosis and GLS dependence [27,137]. SLC7A11-mediated transport of glutamate may deplete the intracellular glutamate/α-KG pool and activate the catabolism of glutamine, leading to more absorption of glutamine [137]. Analogously, high environmental cystine levels or SLC7A11 expression increase the sensitivity of the glutamine inhibitor CB-839 to TCA cycle anaplerosis [68,137].

The amino acid metabolism reprogramming of cancer cells connects proliferation signals with environmental conditions, enabling tumour cells to adapt to changing nutrient levels [138]. mTORC2 is a critical regulator of amino acid metabolism in cancer. It phosphorylates serine 26 at the cytosolic N-terminus of SLC7A11, inhibiting its activity. When micronutrient levels are adequate, glutamate provides carbon and nitrogen sources, and SLC7A11-mediated secretion is disadvantageous to support tumour cell proliferation. Conversely, when there is a lack of nutrition, mTORC2 downregulates SLC7A11 phosphorylation in response to growth factor signalling. Cancer cells protect themselves from cellular stress at least in part by facilitating glutamate efflux and increasing cystine uptake and GSH synthesis.

#### 3.2.4. Tumour Microenvironment

In the tumour microenvironment, SLC7A11-related interactions between immune cells and tumour cells affect tumour survival and proliferation. On the one hand, cytokines secreted by immune cells could affect the expression of SLC7A11 in tumours. Interferon gamma (IFN-γ) secreted by CD8+ T cells downregulates the expression of SLC3A2 and SLC7A11 in tumour cells, impairs the uptake of cystine and, as a consequence, promotes lipid peroxidation and ferroptosis in tumour cells [54]. On the other hand, cysteine competition or glutamate secretion between different immune cells or between immune cells and tumour cells severely affects tumour survival. Cysteine is an essential amino acid for T-cell activation, and plays an important role in tumour monitoring and killing [139]. T cells lack intact SLC7A11 transporters and cystathionases, and rely on neutral amino acid transporters to utilise cysteine exported by APCs. MDSCs express SLC7A11, and only take up cystine, but do not export cysteine. MDSCs compete with APCs for extracellular cystine. In the presence of MDSCs, the release of cysteine by APCs is reduced, thereby limiting the extracellular pool of cysteine and T-cell-activation-mediated antitumour immunity [22,23]. The release of glutamate mediated by SLC7A11 in dendritic cells stimulates constitutively expressed metabotropic glutamate receptor 5 and impairs T-cell activation [140]. Overexpression of SLC7A11 in GBM leads to an increase in extracellular glutamate. This promotes Treg-cell proliferation, activation, and suppressive function and, thus, induces intratumoural immunosuppression [55]. T-cell-induced metabolic changes can affect the fate of GBM cells via SLC7A11, and tumour metabolism also promotes immune evasion by inducing SLC7A11-mediated T-cell activation dysfunction.

SLC7A11 also has potential roles in cancer-associated fibroblasts or vascular remodelling. Cancer-associated fibroblasts (CAFs) are highly dependent on SLC7A11 for protection from exogenous oxidative stress [74], and support pancreatic ductal adenocarcinoma tumour growth and intratumoural fibrosis [141]. High expression of SLC7A11 in CAFs (but not in tumour cells) is an independent prognostic factor for low overall survival. In addition, ATF4 in malignancy of primary brain tumours increases tumour angiogenesis and shapes vascular architecture in an SLC7A11-dependent manner [142]. Unlike primary brain tumours, SLC7A11 reduces choroidal neovascularisation in age-related macular degeneration by inhibiting ferroptosis and vascular endothelial growth factor (VEGF) production [143]. This suggests that the microenvironments of different diseases have different effects on vascular remodelling through SLC7A11.

### 3.3. SLC7A11 vs. Metastasis

SLC7A11 plays an important role in tumour metastasis. Loss of SLC7A11 in melanoma abrogated tumour metastasis in several in vivo murine models of experimental and spontaneous metastasis [144]. Actinidia chinensis (Planch.) increased the accumulation of ROS and ferroptosis by inhibiting SLC7A11 and GPX4 (glutathione peroxidase 4) proteins to suppress the growth and metastasis of gastric cells in a zebrafish xenograft model [145]. The lncRNA Uc.339 inhibited the production of mature miR-339 and regulated SLC7A11, leading to defects in ferroptosis and driving the metastasis of lung adenocarcinoma [146]. PDAC is a highly metastatic tumour with few treatments. The mitochondrial calcium uniporter promotes PDAC cell metastasis by activating the KEAP1-NRF2-SLC7A11 antioxidant axis [147]. The inhibitory effect of miR-139-5p on the proliferation, invasion, and metastasis of PDAC in a murine xenograft model was partly due to its inhibitory effect on the expression of the PI3K/Akt signalling pathway and SLC7A11 [82]. Strikingly, in a murine orthotopic PDAC model utilising human PDAC cells and CAFs, the stable knockdown of SLC7A11 in both cell types—but not in tumour cells alone—reduced tumour metastasis [141]. In addition to genetic and pharmacological approaches to inhibit SLC7A11, SLC7A11 immunotargeted therapies—such as the SLC7A11-targeted DNA vaccination [108], a virus-like particle immunotherapy targeting the SLC7A11 protein [109], and an anti-SLC7A11 viral vaccine based on the bovine herpesvirus 4 vector [110]—also impeded the development of lung metastasis in murine xenograft models of human breast cancer.

In fact, glutamate secretion by SLC7A11 may also promote the intrinsic invasiveness of cancer cells. Glutamate promotes invasive behaviour in breast cancer cells by activating metabotropic glutamate receptors and upregulating membrane type 1 metalloprotease to disrupt the basement membrane [148]. Glutamate secretion is induced by IL-1β, upregulated programmed death ligand 1 (PD-L1), and colony-stimulating Factor 1 (CSF1) through the α-KG/HIF1α axis, which promotes hepatic cell carcinoma (HCC) metastasis [149]. Pharmacological interference with the release of glutamate from cancer cells was found to potentially limit the host bone response to invading tumour cells and impair bone metastasis in a murine xenograft model of human breast cancer [150].

### 3.4. SLC7A11 vs. Therapeutic Resistance

The mechanism of SLC7A11 in therapeutic resistance involves the antioxidant stress system, ferroptosis, nutrient limitation, autophagy, multidrug resistance, etc. Microarray analysis of the gene expression of transporter proteins in 60 human cancer cells showed that SLC7A11 expression was correlated with the efficacy of 1400 candidate anticancer drugs [151]. SLC7A11 is positively correlated with L-alanosine by increasing cellular uptake. L-alanosine is an amino acid analogue that has anticancer activity. The expression of SLC7A11 provides GSH maintenance by supplying cystine, and is negatively correlated with the potency of anticancer drugs, such as geldanamycin. It can be seen that SLC7A11 has a complex influence on the potency of antitumour drugs, and that it hinges upon different situations.

#### 3.4.1. Antioxidant Function and Ferroptosis

After chemotherapy or radiotherapy, some cancer cells may upregulate SLC7A11 expression to resist oxidative stress, inhibit ferroptosis, and develop therapeutic resistance. Nuclear NRF2 and SLC7A11 were overexpressed in oesophageal squamous-cell carcinoma tissues, and this overexpression inhibited ferroptosis and induced radioresistance [69]. The expression of SLC7A11 in GBM [152,153], CD133-positive hepatocellular carcinoma cells [154], poorly differentiated liver cancer tissues [92], and metastatic and/or recurrent urothelial carcinoma [93] was downregulated via multiple mechanisms of interference to inhibit GSH synthesis, induce intracellular ROS levels, and increase sensitivity to chemotherapy drugs. However, the response to SLC7A11 depletion is cell-type-dependent. CD44 knockdown reduced the protein stability of SLC7A11, but increased GSH with enhanced chemoresistance through NRF2/AP-1-mediated upregulation of glutamate–cysteine ligase in malignant mesothelioma cells [155]. A recent study showed that radioresistance in HCC may be associated with Cu-Fe homeostasis involved in SLC7A11. Radiotherapy could induce Cu accumulation, inhibit the ubiquitin degradation of HIFα, and promote the transcription of SLC7A11, inhibiting ferroptosis [156].

#### 3.4.2. Nutrient Dependency

Cancer cells with higher SLC7A11 expression levels may be highly dependent on certain nutrients—such as glucose and glutamine—for survival, which can inform therapeutic strategies to target these cancer-specific metabolic vulnerabilities. Under glucose restriction, the overexpression of SLC7A11 significantly decreases the mismatch repair gene, shows an increased level of double-strand breaks, and increases sensitivity to radiotherapy in GBM cells [157]. Furthermore, the cytotoxicity of the SLC7A11 inhibitor sulfasalazine in CD44v-expressing stem-like head and neck squamous cells is related to glutamine uptake and GDH-related α-KG production. The ablation of GDH reduces drug sensitivity to SASP [158].

#### 3.4.3. Autophagy

In contrast, SLC7A11 was significantly decreased in 90 drug-resistant ovarian cancer tissues compared with their controls, conferring drug resistance by inhibition of cell autophagy as a competing endogenous RNA. Upregulation of SLC7A11 significantly increased the sensitivity of ovarian cancer cells to paclitaxel in vitro [159].

#### 3.4.4. Multidrug Resistance

The drug resistance of breast cancer or non-small-cell lung cancer cells developed following long-term application of doxorubicin or docetaxel. Compared with the corresponding sensitive cells, SLC7A11 expression was lower in two drug-resistant cell lines [34,97]. The multidrug-resistant protein P-glycoprotein (P-gp) is one of the most important defence mechanisms of tumour cells against the actions of drugs. Downregulation of SLC7A11 or cystine deprivation significantly enhanced ROS-induced overexpression of P-gp in breast cancer cells and the resistant subline derived from them. In contrast, overexpression of SLC7A11, adequate cystine supplementation, or treatment with N-acetylcysteine (ROS scavenger) significantly reduced the expression and activity of P-gp [34]. Cystine supplementation increased sensitivity to chemotherapeutic drugs in a drug-resistant tumour-bearing murine model of lung cancer [97]. It can be inferred that ROS and SLC7A11 are two related factors affecting P-gp expression and function, and SLC7A11 may be a potential target for regulating P-gp-associated drug resistance.

## 4. Conclusions

Over the years, numerous studies on SLC7A11 have given us a deeper understanding of its basic functions, regulation, and pathophysiological effects, but whether SLC7A11 is a friend or foe of cancer remains a controversial issue.

Some authors have demonstrated that SLC7A11 is overexpressed in various types of tumour cells, maintains intracellular redox balance, and inhibits intracellular ROS accumulation and ferroptosis resistance [77,94,123]. Downregulation of SLC7A11 expression or inhibition of SLC7A11 function was expected to increase the amount of intracellular ROS and trigger cell death [8,91,103,107,131]. In addition, SLC7A11-related interactions between immune cells and tumour cells affect tumour survival and proliferation. CD8+ T cells secreted IFN-γ, which could downregulate SLC7A11 expression and affect tumour cell growth [54]. Cysteine is required for T-cell activation, and can only be obtained from the tumour microenvironment, because T cells do not have intact SLC7A11 [22,23]. Glutamate secreted by SLC7A11 of tumour cells inhibits T-cell activation [140] and stimulates the proliferation of Treg cells to induce intratumoural immunosuppression [55].

Other authors have proposed that cancer cells require large amounts of energy substrates to maintain a strong antioxidant defence system through SLC7A11, resulting in glucose and glutamine dependence. Glucose metabolism provides large amounts of NADPH for the reduction of cystine to cysteine [70,132]. SLC7A11 transports glutamate out of the cell and activates the catabolism of glutamine, leading to the negative feedback absorption of more glutamine [51]. Cancer cells with high SLC7A11 expression are more sensitive to glucose and glutamine restrictions due to their nutrient dependence. Inhibition of SLC7A11 expression facilitates tumour adaptation to a hypoxic microenvironment [98]. The depletion of cysteine promotes the inactivation of EglN1 and the accumulation of intracellular HIF1α.

Alternatively, it has been suggested that a moderate increase in ROS is conducive to cell proliferation and survival, and can drive the occurrence and invasion of malignant tumours and the development of drug resistance in tumours. Due to excessive oxidative stress and inflammation, SLC7A11-deficient mice showed an increased incidence of chemo-induced tumourigenesis [120]. Under the stimulation of chemotherapeutic drugs, the expression of SLC7A11 in tumour cells is decreased, ROS are increased, and the expression of multidrug-resistant proteins is increased, causing drug resistance [34,97]. Interestingly, overexpression of SLC7A11 enhances the antioxidant ability of some resistant cancer cells to overcome drug-induced oxidative stress and ferroptosis [69,91,151]. The possible cause of this seemingly contradictory phenomenon is the concentration and duration of ROS exposure. There may be a toxicity threshold for ROS to induce tumour cell death. Only high levels of ROS can induce cell death, and ROS levels below the threshold increase the degree of tumour malignancy [160].

Based on the above complex role of SLC7A11 in cancer, we believe that individualised detection of tumour genotypes should be performed when targeting SLC7A11 for tumour therapy. For cancer cells with high SLC7A11 expression, one therapeutic option is to use SLC7A11-targeted inhibitors—such as SASP—to directly inhibit SLC7A11-mediated cystine uptake and destroy antioxidant defence. The other therapeutic option is to use glucose transporters or glutaminase inhibitors to take advantage of the metabolic vulnerability of cancer cells to limit their energy intake. However, for cancer cells with low SLC7A11 expression, the situation is more complicated. In the case of cell proliferation, invasion and drug resistance caused by appropriate ROS, cystine supplementation, or SLC7A11 activation could rebalance redox and reduce the effect of ROS on the malignancy of tumours. In addition, low SLC7A11 expression may also be a metabolic vulnerability of cells, and further inhibition of SLC7A11 may also inhibit tumour growth or survival.

In conclusion, the SLC7A11 gene is involved in different stages of cancer development. SLC7A11 may be a potential target for cancer therapy. However, its role depends on the specific situation, and there is still a long way to go before it can be translated into the clinical setting.

## Figures and Tables

**Table 1:** A list of drugs reported as inhibitors/inducers of SLC7A11.

Compound	Inhibitor/Inducer	Drug Group	Cancer Type
Acetylcysteine	Inducer	Approved	-
Riluzole	Inhibitor	Approved	MEL [100]
Sulfasalazine	Inhibitor	Approved	SAR [103], GC [111], PC [112], GBM [113], LUAD [114]
Erastin	Inhibitor	Experimental	FSA [76], GC [33], PC, LUAD, HNSC [115]
Lanperisone	Inhibitor	Approved	FSA [105]

MEL: melanoma; SAR: sarcoma; GC: gastric cancer; PC: prostate cancer; GBM: glioblastoma; LUAD: lung cancer; HNSC: head and neck squamous-cell carcinoma; FSA: fibrosarcoma.

## Data Availability

Not applicable.
